# VmPacC-mediated pH regulation of *Valsa mali* confers to host acidification identified by comparative proteomics analysis

**DOI:** 10.1007/s44154-023-00097-y

**Published:** 2023-06-21

**Authors:** Liangsheng Xu, Hailong Liu, Shan Zhu, Yangguang Meng, Yinghao Wang, Jianyu Li, Feiran Zhang, Lili Huang

**Affiliations:** State Key Laboratory of Crop Stress Biology for Arid Areas, College of Plant Protection, Northwest A&F University, Yangling, Shaanxi 712100 China

**Keywords:** *Valsa mali*, TMT, PacC, Proteomics, Differentially expressed protein

## Abstract

Apple valsa canker caused by the Ascomycete fungus *Valsa mali* is one of the most serious diseases of apple, resulting in huge economic losses in the apple-growing area of China. Previous study found that the pathogen could acidify the infected tissues to make lower ambient pH (from 6.0 to 3.5) for their successfully colonization. The pH signaling transcription factor *VmPacC* is required for acidification of its environment and for full virulence in *V. mali*. It is known that the functional cooperation of proteins secreted by *V. mali* plays pivotal role in its successful colonization of host plants. In this study, we used tandem mass tag (TMT) labeling coupled with LC-MS/MS-based quantitative proteomics to analyze the *VmPac*C-mediated pH regulation in *V. mali,* focusing on differentially expressed proteins (DEPs). We identified 222 DEPs specific to *VmPacC* deletion, and 921 DEPs specific to different pH conditions (pH 6.0 and 3.4). Gene Ontology (GO) and Kyoto Encyclopedia of Genes and Genomes (KEGG) pathway analyses indicated that these DEPs were mainly involved in pathways associated with carbon metabolism, biosynthesis of antibiotics, citrate cycle (TCA cycle), glycolysis/gluconeogenesis, glutathione metabolism, ribosomes, and pentose phosphate pathways. Additionally, we identified 119 DEPs that were shared among the *VmPacC* deletion mutant and different pH conditions, which were mainly related to energy metabolism pathways, providing the energy required for the hyphal growth and responses to environmental stresses. A protein-protein interaction (PPI) network analysis indicated that most of the shared proteins were mapped to an interaction network with a medium confidence score of 0.4. Notably, one uncharacterized protein (KUI69106.1), and two known proteins (heat shock protein 60 (KUI73579.1), aspartate aminotransferase (KUI73864.1)) located in the core of the network were highly connected (with ≥ 38 directed edges) with the other shared DEPs. Our results suggest that *VmPacC* participates in the pathogen’s regulation to ambient pH through the regulation of energy metabolism pathways such as the glycolysis/gluconeogenesis pathway and TCA cycle. Finally, we proposed a sophisticated molecular regulatory network to explain pH decrease in *V. mali*. Our study, by providing insights into *V. mali* regulating pH, helps to elucidate the mechanisms of host acidification during pathogen infection.

**Supplementary Information:**

The online version contains supplementary material available at 10.1007/s44154-023-00097-y.

## Introduction

The survival and reproduction of pathogenic fungi depend on the ability to produce adaptive or protective responses to their immediate environmental conditions. The pH, a crucial physiochemical factor, is one of the most important environmental parameters affecting the survival and growth of the pathogens (Lund et al. 2020). The fluctuations in ambient pH significantly impacts the enzyme activities, cell-wall remodeling, extracellular nutrient availability, and protein synthesis of pathogenic fungi (Alkan et al. 2013; Li et al. 2022, 2010; Selvig and Alspaugh 2011; Hu et al. 2020). Regulation to ambient pH is therefore essential for pathogenic fungi to successfully infect their host (Cooney and Klein 2008; Prusky and Yakoby 2003).

To survive under the varied pH conditions, fungal pathogens have evolved a complicated and conserved system, the so-called Pal-pH pathway, to regulate the expression of genes involved in the fungal response to ambient pH (Tilburn et al. 1995; Vylkova 2017; Penalva et al. 2008). For pathogenic fungi, the Pal-pH pathway not only enables fungi to thrive over a wide pH range, but also determines whether they can successfully invade and colonize the target host. The Rim101/PacC zinc-finger transcription factor is the terminal component of the Pal-pH pathway that controls the expression of both acid- and alkaline-expressed genes in *Aspergillus* and other fungus species (Tilburn et al. 1995; Penalva et al. 2008). PacC activates or represses different target genes during cellular responses to ambient pH (Luo et al. 2017). For example, at alkaline ambient pH, *PacC* of *Aspergillus* activates the transcription of alkaline-expressed genes and represses transcription of acid-expressed genes (Tilburn et al. 1995). In *Fusarium graminearum*, the PacC homologue Pac1 negatively regulates *Tri* gene expression and toxin production (Merhej et al. 2011). More importantly, there is abundant evidence indicating that *PacC* exists in various fungal species and plays important roles in the regulation of pathogenicity. Deletion of PacC homologs reduced virulence in most pathogenic fungi, including *Colletotrichum gloeosporioides* (Yakoby et al. 2000), *Penicillium digitatum* (Zhang et al. 2013), *Magnaporthe oryzae* (Landraud et al. 2013), *Sclerotinia sclerotiorum* (Rollins 2003), and *Valsa mali* (Wu et al. 2018). On the other hand, PacC was confirmed to be a negative regulator of virulence in *F. oxysporum* (Caracuel et al. 2003) and *F. graminearum* (Merhej et al. 2011), indicating that there was a complex regulatory network of PacC to affect virulence in different fungal pathogens.

*V. mali*, the causal agent of apple Valsa canker, is a necrotrophic fungus that causes severe necrosis on apple trees. In Eastern Asia, *V. mali* causes substantial yield losses to apple farms each year (Ke et al. 2013). During infection, the pathogen was found to acidify the infected tissues by secreting citric acids leading to the reduced ambient pH (from 6.0 to 3.5). The PacC ortholog *VmPacC*, by affecting the citric acid generation and accumulation, is an essential virulence factor for *V. mali* (Wu et al. 2018). In addition, genomic and transcriptomic analyses of *V. mali* revealed that genes associated with enzymatic hydrolysis, secondary metabolite synthesis, and acid secreted proteases, which might be the main virulence factors of adaptive infection, significantly expanded in the genome (Yin et al. 2015; Ke et al. 2014). These findings provide important clues for further investigations to unveil the modes of PacC regulation of pathogenicity in *V. mali*.

Proteins participate in organism growth and development, secondary metabolism, and many other biological processes, reflecting the response of various physiological functions to stress (Ball et al. 2020; Zhang et al. 2019). Quantitative proteomics focuses on screening and identifying proteome variations among different states of an organism, i.e., revealing and verifying changes in proteomics (Pechanova et al. 2013; Bai et al. 2021). At present, tandem mass tag (TMT) technology is widely used in the mass spectrometry (MS) analysis of differentially expressed proteins (DEPs) due to its advantages in accurate quantification, good repeatability, and high sensitivity (Gonzalez-Fernandez and Jorrin-Novo 2012; Han 2017).

In this study, we used an integrated approach involving high-throughput TMT protein labeling coupled with LC-MS/MS-based quantitative proteomics to screen for DEPs that reveal how *VmPacC* participates in pH regulation in *V. mali*. GO and KEGG pathway analyses were performed to discern the main functions of these proteins and predict their possible relationships with each other. Finally, we proposed a model of *V. mali* response to ambient pH based on the results of the proteomics analysis to inform and guide further research with a basic understanding of the pathogenesis of *V. mali*.

## Results

### DEPs from apple twigs infected with the wild-type strain and the *VmPacC* deletion mutant (*ΔVmPacC*) of *V. mali*

To identify the pathogenicity-related proteins regulated by *VmPacC* in *V. mali*, the healthy apple twig tissues and diseased tissues inoculated with the wild-type strain and *VmPacC* deletion mutant were collected (Fig. S1) (Wu et al. 2018). Four corresponding protein libraries, named WT-H, WT-V, PacC-H, and PacC-V, were constructed, and subjected to a TMT-based quantitative proteomic analysis. Proteins were identified as DEPs based on predefined cut-offs for FC > 1.3 for up-regulation or < 0.77 for down-regulation and *p* < 0.05 significance.

**Fig. 1:**
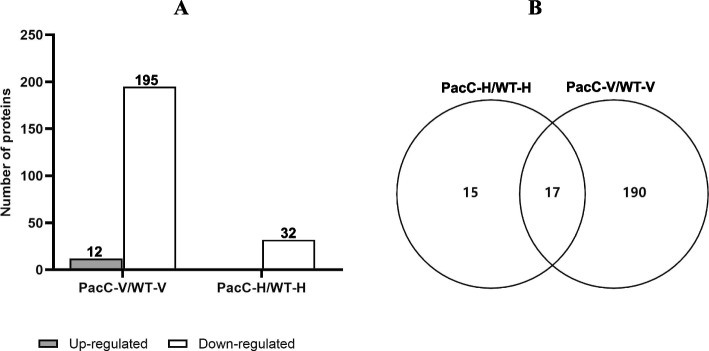
Differentially expressed proteins (DEPs) in *VmPacC* deletion treatment. **A** Numbers of the DEPs detected in *VmPacC* deletion treatment. **B** Venn diagram showing commonly expressed (shared) proteins and differentially expressed proteins in the PacC-H versus WT-H and the PacC-V versus WT-V comparison groups. H, healthy tissue; V, diseased tissue

As shown in Fig. 1A, expression of 32 proteins were down-regulated in the PacC-H with respect to the WT-H group (PacC-H/WT-H group comparison) (Fig. 1A). For the PacC-V/WT-V comparison, a total of 207 DEPs were identified, with 12 proteins up-regulated and 195 down-regulated. These data indicated that the DEPs regulated by *VmPacC* were directly or indirectly involved in fungal pathogenic processes, they were considered potential virulence factors. Additionally, we found that there were 17 proteins shared between the PacC-H/WT-H DEPs and PacC-V/WT-V DEPs (Fig. 1B), which included 9 ribosomal proteins and 8 basic metabolic proteins (Table S1). This finding suggested that these common proteins were regulated by *VmPacC* during the process of *V. mali* infecting the apple bark and contributed to the growth and development of the mycelium. In addition, there were 12 up-regulated and 210 down-regulated proteins identified (total of 222 DEPs, combination of PacC-H/WT-H and PacC-V/WT-V) in the PacC group for the PacC versus WT group comparison (Fig. 1B). These proteins were identified as ones regulated by PacC and played an important role during infection.

**Fig. 2:**
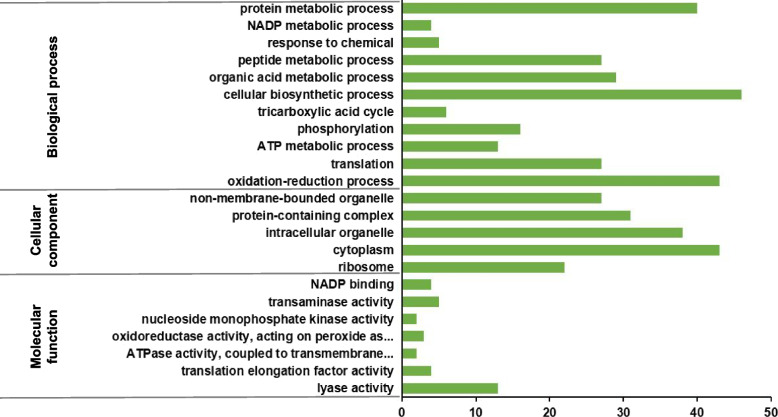
Gene ontology (GO) analysis of differentially expressed proteins (DEPs) in the PacC versus WT comparison. GO annotation for 222 DEPs in three categories: biological processes, cellular components, and molecular functions. The abscissa represents the number of proteins corresponding to the function. Green columns represent the number of proteins by category

To further reveal the functional characteristics of DEPs regulated by *VmPacC*, we conducted a GO functional annotation in the PacC versus WT group comparison. The GO annotation assigned the 222 DEPs to 296 GO terms (*p* < 0.05), including 168 biological processes, 35 cellular components and 93 molecular function terms (Table S2). The most common GO annotation terms are shown for the three categories in Fig. 2. The DEPs in the biological processes (BP) category included proteins associated with process in cellular biosynthetic, oxidation-reduction, protein metabolic, organic acid metabolic, peptide metabolic, translation, phosphorylation, etc. DEPs in the MF category included proteins associated with lyase activity, transaminase activity, NADP binding, etc. (Fig. 2). Under the cell component (CC) category, the most abundant groups included the cytoplasm, intracellular organelle, protein-containing complex, non-membrane-bounded organelle, ribosome, etc. (Fig. 2).

**Fig. 3:**
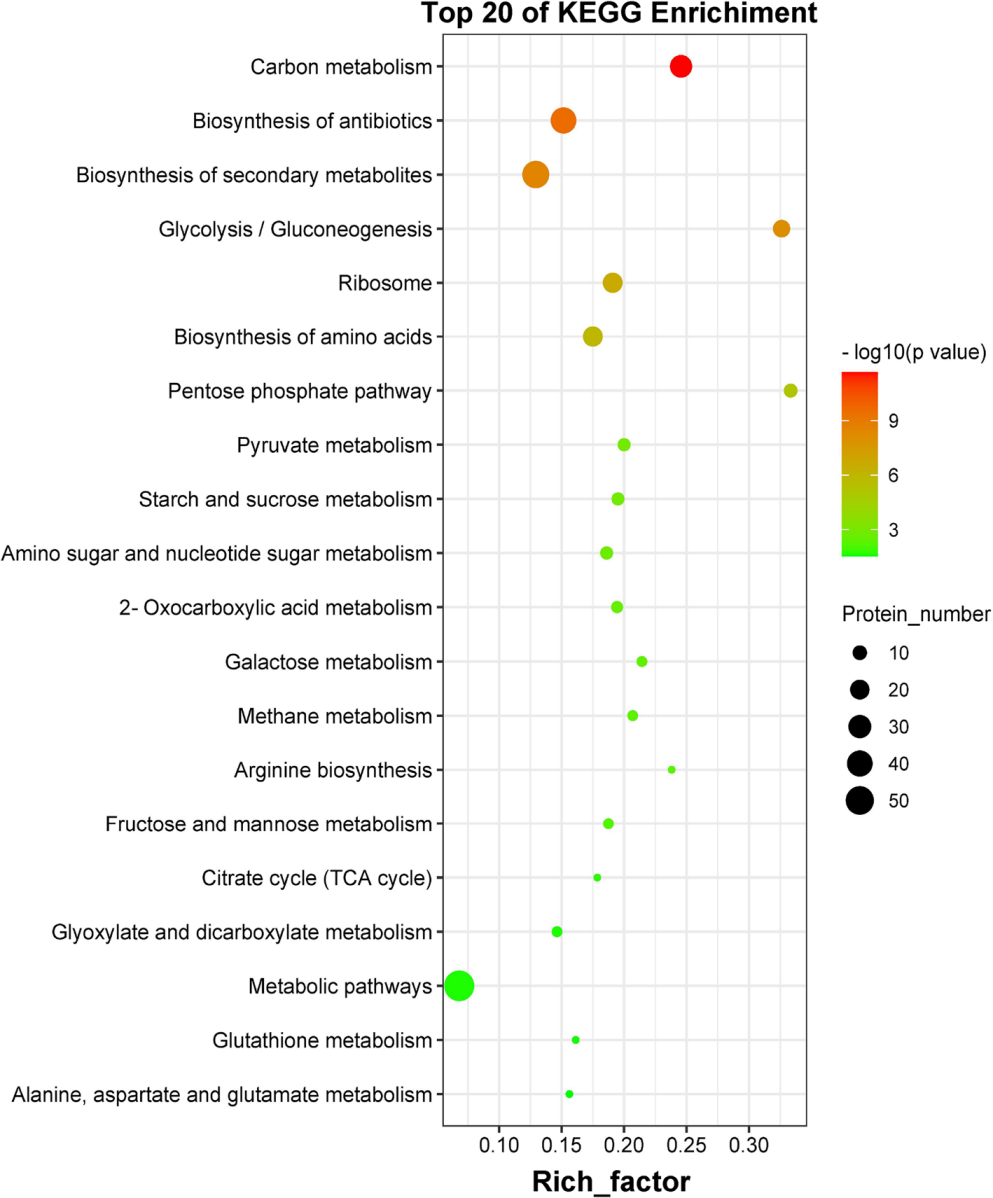
Kyoto Encyclopedia of Genes and Genomes (KEGG) pathway enrichment analysis of the 222 DEPs in the PacC versus WT comparison. The abscissa represents the enrichment factor, and the ordinate represents the KEGG classification of the metabolic pathway. Circles indicate numbers of enriched proteins, and colors depict the *p*-value. The size of each circle represents the number of significant DEPs enriched in the corresponding pathway. The enrichment factor was calculated using the number of enriched DEPs divided by the total number of background proteins in the corresponding pathway

We conducted a KEGG pathway analysis to explore the metabolic pathways and functions of the 222 DEPs in the PacC/WT comparison. The results revealed that the 222 DEPs were associated with 72 enriched metabolic pathways, 23 of which were significantly altered (*p* < 0.05) (Table S3). The top 20 enriched pathways are shown in Fig. 3. DEPs were highly clustered in pathways of signaling (such as carbon metabolism), biosynthesis (of antibiotics, secondary metabolites, and amino acids), glycolysis/gluconeogenesis, ribosome, pentose phosphate, pyruvate metabolism, starch and sucrose metabolism, etc. (Fig. 3). These results indicated that *VmPacC* may exert its function through a broad spectrum of pathways.

### DEPs under different ambient pH of *V. mali*

In this experiment, we maintained buffered culture media at pH 6.0 or pH 3.4 to mimic pH values of bark tissues and investigated the effects of the two different ambient pH values on the adaptive regulation of *V. mali*. We sampled intracellular proteins by harvesting the mycelium, and extracellular proteins by collecting the filtrates, and maintained the samples on YEPD media buffered at pH 6.0 and pH 3.4 for 24 h.

**Fig. 4:**
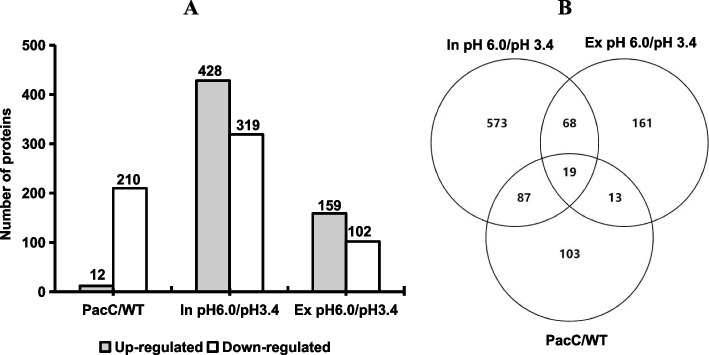
Differentially expressed proteins (DEPs) in *VmPacC* deletion treatment and different pH conditions. **A** Numbers of the DEPs detected in *VmPacC* deletion treatment and different pH conditions. **B** Venn diagram of commonly expressed (shared) proteins and differential expressed proteins (DEPs) in the PacC versus WT and the pH 6.0 versus pH 3.4 comparison groups

Quantitative whole-proteome profiling of *V. mali* maintained under two pH conditions specifically identified a total of 1008 pH-responsive proteins (87 proteins were shared in intracellular and extracellular) based on the predefined cut-off fold changes for down-regulation (FC < 0.77) and up-regulation (FC > 1.3) with *p* < 0.05 significance (Fig. 4A, Table S4). Of the 1008 proteins 428 were up-regulated and 319 were down-regulated intracellularly, while 159 proteins were up-regulated and 102 were down-regulated extracellularly (Fig. 4A). Overall, the 1008 - 87 = 921 proteins that were regulated by pH made up about 8% of the 11,261 predicted proteins, indicating that *V. mali* had a wide-ranging proteomic response to external pH.

**Fig. 5:**
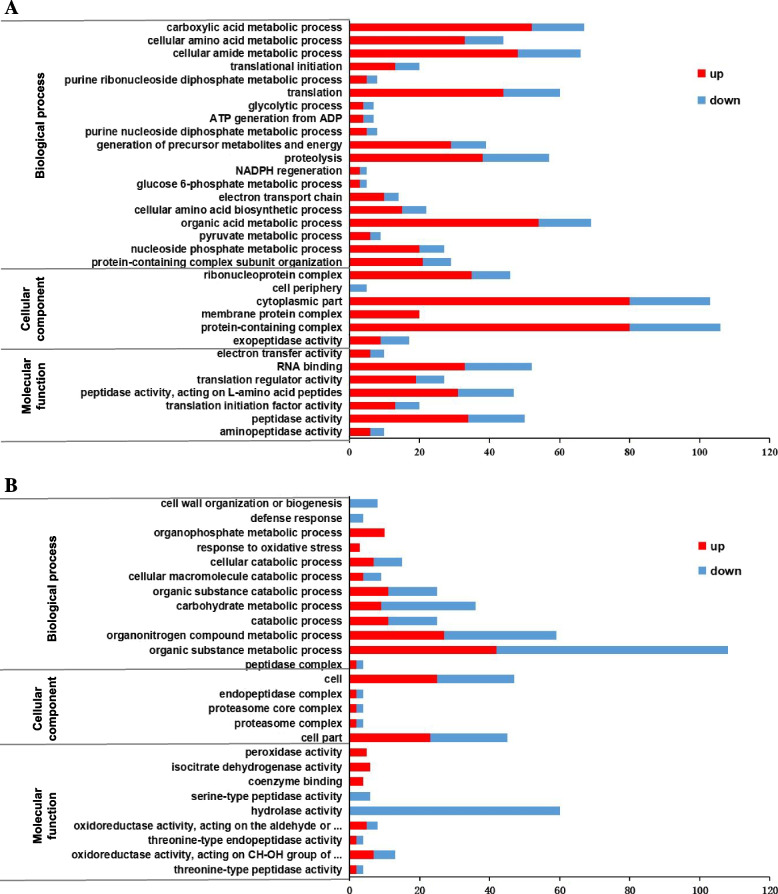
Gene Ontology (GO) annotation analysis for the DEPs under different ambient pH of *V. mali.***A** Intracellular DEPs. **B** Extracellular DEPs. DEPs were annotated in three categories: biological processes, cellular components, and molecular functions. The abscissa represents the number of proteins corresponding to the function. Red columns represent up-regulated proteins, blue columns represent down-regulated proteins

To further explore DEPs in the intracellular and extracellular domain as separate components of the response to different ambient pH, the intracellular DEPs and extracellular DEPs were separately annotated using GO analysis (Fig. 5).

Among the intracellular DEPs (Fig. 5A), the top five most enriched annotated GO terms under the BP category for up-regulated proteins included organic acid metabolic process, carboxylic acid metabolic process, cellular amid metabolic process, translation, and proteolysis. By contrast, for down-regulated proteins, protein metabolic process was the most abundantly enriched BP term. Under CC, the most enriched GO terms for both up-regulated and down-regulated proteins were cytoplasmic part, protein-containing complex, and ribonucleoprotein complex. Under MF, the top enriched GO terms for both up-regulated and down-regulated proteins were RNA binding and peptidase activity (Fig. 5A).

The same analysis was repeated for extracellular DEPs (Fig. 5B). Under the cellular component (CC) category, the same terms were enriched by up-regulated and down-regulated proteins, with cell and cell part being the top two enriched terms. However, under both the MF and BP groups, there were some differences between the up-regulated and down-regulated proteins. Under MF, the most abundantly annotated GO term included oxidoreductase activity, isocitrate dehydrogenase activity, and peroxidase activity for up-regulated proteins, whereas the top terms included hydrolase activity and serine-type peptidase activity for down-regulated proteins. Under BP, the top two annotated GO terms for up-regulated proteins included organic substance metabolic process and organonitrogen compound metabolic process. By comparison, there were three annotated GO terms for down-regulated proteins, namely, organic substance metabolic process, organonitrogen compound metabolic process, and carbohydrate metabolic process.

**Fig. 6:**
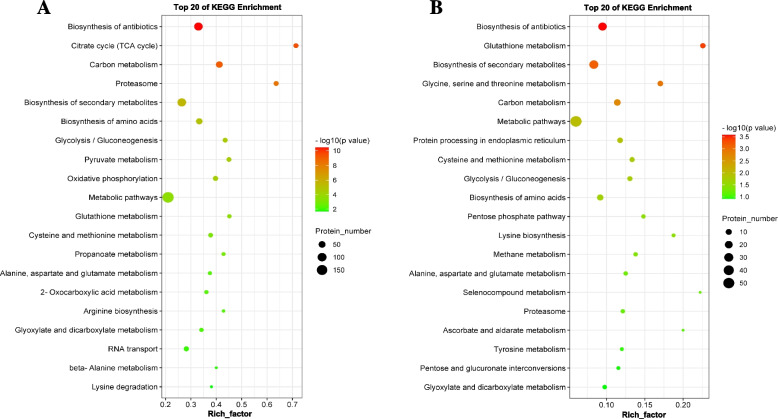
KEGG enrichment analysis separately for **A** intracellular and **B** extracellular differentially expressed proteins (DEPs) under different ambient pH of *V. mali*. The enrichment factor (abscissa) is shown for each KEGG-classified metabolic pathway (ordinate). Circles indicate the number of enriched proteins, and colors depict the *p*-value. The size of each circle represents the number of significant DEPs enriched in the corresponding pathway. The enrichment factor was calculated using the number of enriched DEPs divided by the total number of background proteins in the corresponding pathway

To further analyze the key enriched metabolic pathways in the *V. mali* response to ambient pH, a KEGG pathway enrichment analysis was performed. For the 747 intracellular DEPs, we have found 104 enriched metabolic pathways, 28 of which were significantly altered (*p* < 0.05). The top 20 enriched pathways for the intracellular DEPs (Fig. 6A) included biosynthesis of antibiotics, citrate cycle (TCA cycle), carbon metabolism, proteasome, biosynthesis of secondary metabolites and amino acids, glycolysis/gluconeogenesis, pentose phosphate pathway, pyruvate metabolism, oxidative phosphorylation, glutathione metabolism, etc. The 258 extracellular DEPs were mapped to 76 enriched metabolic pathways, 13 of which were significantly altered (*p* < 0.05) (Fig. 6B), including biosynthesis of antibiotics, glutathione metabolism, biosynthesis of secondary metabolism, amino acid (glycine, serine, and threonine) metabolism, carbon metabolism, etc. These results indicated that the pH value of the surrounding environment was decreased by the secretion of organic substances in the process of the acidification of the wooden bark.

### pH-responsive proteins regulated by PacC

We further investigated the PacC-regulated pH-responsive proteins by comparing the proteins common to the PacC and WT groups versus those common to the pH 6.0 and the pH 3.4 groups. This comparison identified a total of 119 shared proteins for these groups (Fig. 4B). Most of the shared proteins were up-regulated under high pH conditions and down-regulated in the *VmPacC* deletion mutant treatment (Fig. S2). This suggested that these proteins were synthesized and secreted by *V. mali* after sensing environmental pH signals to maintain intracellular pH stability and justify the environmental pH.

**Fig. 7:**
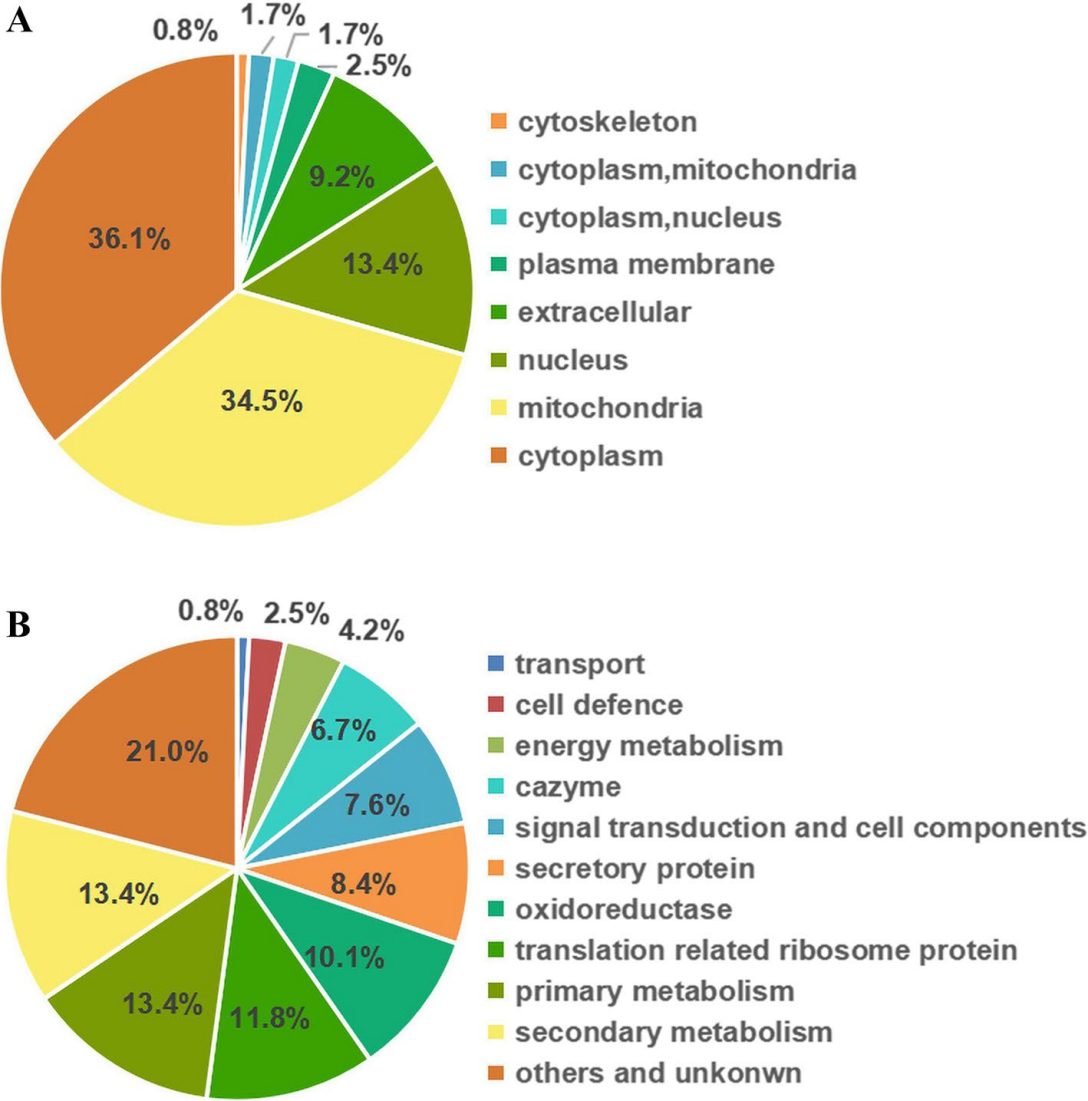
Analysis of the 119 shared proteins in the PacC versus WT and the pH 6.0 versus pH 3.4 comparison groups. **A** Predicted cellular location of the proteins. **B** Functional annotation of the proteins

All 119 identified shared proteins were categorized according to their subcellular localizations, revealing that these proteins were concentrated in the cytoplasm (36%), mitochondria (34%), nucleus (13%), extracellular medium (9%), plasma membrane (3%) and others (9%) (Fig. 7A). The 119 shared proteins were also classified into 11 functional groups, including primary metabolism (13.4%), secondary metabolism (13.4%), translation related ribosome protein (11.8%), oxidoreductase (10.1%), secretory protein (8.4%), signal transduction and cell components (7.6%), cazyme (6.7%), energy metabolism (4.2%), cell defense (2.5%), transport (0.8%), and others and unknown (21%) (Fig. 7B). These results indicate, above all else, that pH regulation mediated by the zinc-finger transcription factor PacC may depend on a series of signal cascades reactions. The PacC mainly respond to adverse environmental conditions by regulating energy metabolism pathways and controlling the secretion of secondary metabolism products.

### Analysis of PPI networks

**Fig. 8:**
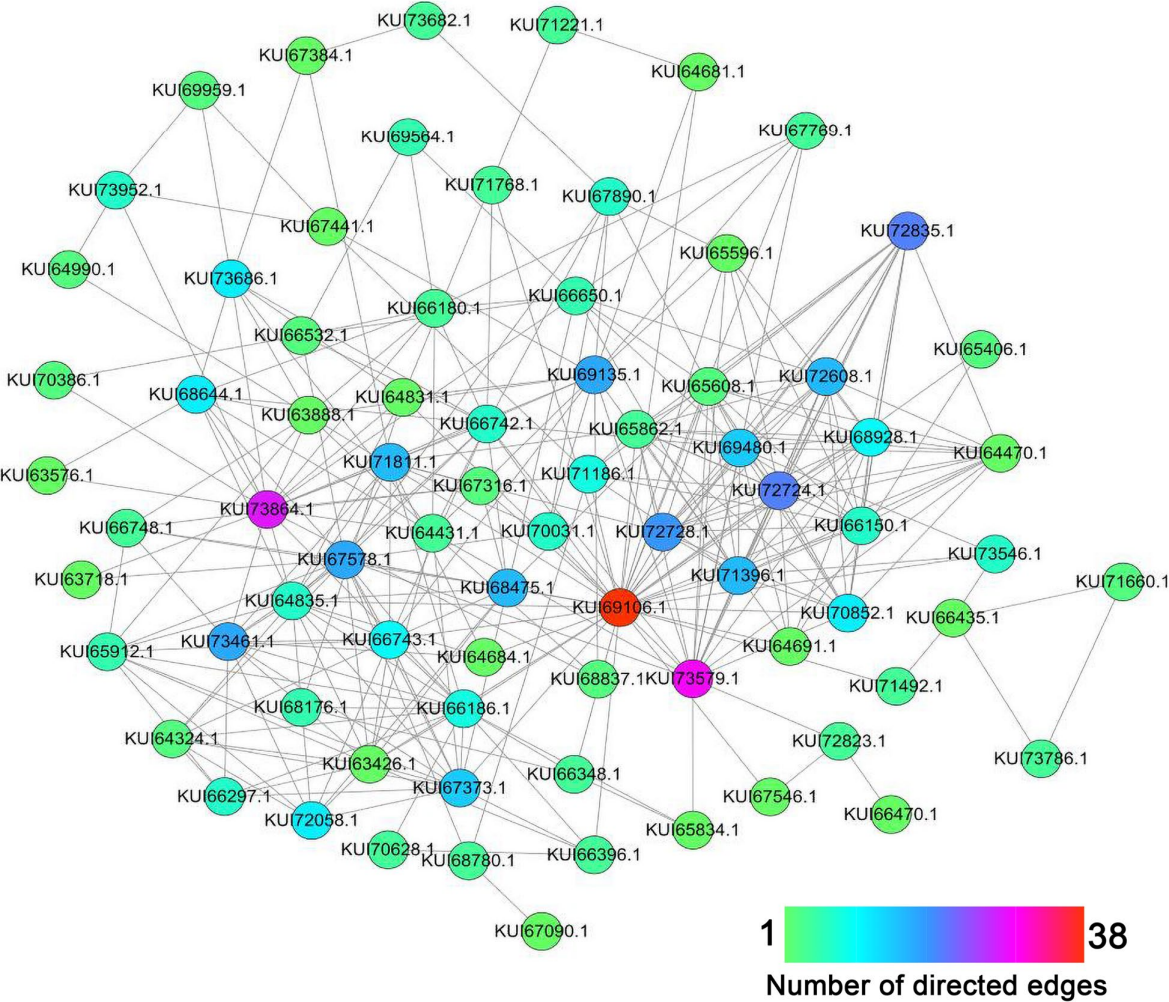
Protein-Protein Interaction (PPI) network of the 119 selected shared proteins based on STRING analysis. The network nodes represent 77 differentially expressed proteins (DEPs). Each node in the network represents a DEP. Directed edges represent interactions between two proteins. These DEPs are listed in Table S4

STRING analysis was used to explore the potential interaction network of the 119 proteins shared between the PacC versus WT and pH 6.0 versus pH 3.4 comparison groups. As shown in Fig. 8, 77 of the 119 shared proteins were mapped to an interaction network with a medium confidence score of 0.4. Among them, an uncharacterized protein (KUI69106.1), heat shock protein 60 (KUI73579.1), and aspartate aminotransferase (KUI73864.1) were in the core of the network and linked to many other DEPs. Notice that protein KUI69106.1, whose functions are still unclear, was positioned in the core of the network and connected with 38 other proteins, suggesting it has important roles in the response of *V. mali* to environmental pH. Also notice that the remaining 119 - 77 = 42 proteins that were identified in the above analysis as to play an important role in the process of *V. mali* response to stress were unconnected with the 77 proteins in the network because their functions were either unrelated or unknown. The 119 shared proteins are summarized with details in Table S5.

### qRT-PCR validation of DEPs

**Fig. 9:**
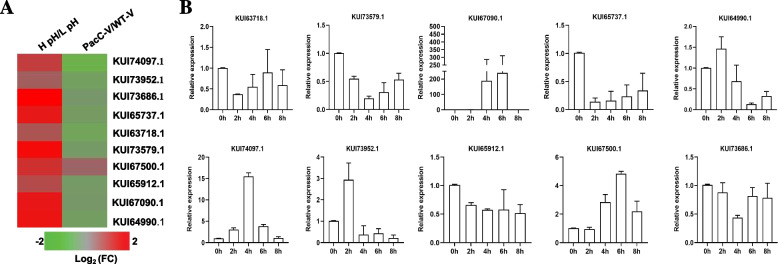
Ten proteins selected for qRT-PCR in the PacC versus WT (PacC-V/WT-V) and pH 6.0 versus pH 3.4 (HpH/LpH) comparison groups. **A** Heat map of 10 DEPs in the H pH/L pH and PacC-V/WT-V comparison groups. **B** Relative transcriptomic analysis of mRNA for the 10 genes encoding the above 10 shared proteins using qRT-PCR

To verify DEPs at the level of transcription, the genes of encoding the 10 shared proteins between the PacC and WT groups and between the pH 6.0 and pH 3.4 groups were selected for a quantitative reverse transcription polymerase chain reaction (qRT-PCR) assay. The 10 proteins included aldehyde dehydrogenase (KUI64990.1), phospholipase D2 (KUI63718.1), acetyl-CoA hydrolase (KUI73952.1), beta-cyclopianiate dehydrogenase (KUI67500.1), heat shock protein 60 (KUI73579.1), citroyl synthetase (KUI73686.1), arginase (KUI65737.1), glucose-6-phosphate 1-dehydrogenase (KUI65912.1), versicolorin reductase (KUI74097.1), putative NADPH-dependent methylglyoxal reductase GRP2 (KUI67090.1). While the expression levels for all 10 shared proteins were higher under high pH than low pH (Fig. 9A), the qRT-PCR assay results showed that the mRNA transcription levels (Fig. 9B) were inconsistent with the TMT identification results. Furthermore, a total of 27 DEPs were found to overlap with the co-expression network that had been identified through transcriptome profiling data generated by Ke et al (2014) (Table S6). However, poor correlation between transcriptional changes and translation dynamics were found. It is well known that gene transcription and protein abundance are poorly correlated (Maier et al. 2009). Thus, further experimental verification was required to establish a correlation between transcriptome and proteome.

### Proteomics validation

**Fig. 10:**
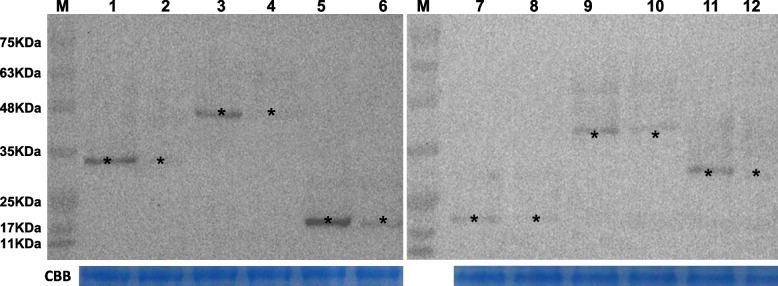
Partial DEPs were chosen for validation using Western blotting. Lane M, prestained protein mass markers; Lane 1-2, KUI73786; Lane 3-4, KUI69959; Lane 5-6, KUI63935; Lane 7-8, KUI65608; Lane 9-10, KUI72794; Lane 11-12, KUIKUI67890. Line 1, 3, 5, 7, 9, 11 represented high pH (pH 6.0) group; line 2, 4, 6, 8, 10, 12 represented low pH (pH 3.4) group. Black asterisks indicate the protein bands of interest

To ensure the reliability of the quantitative proteomics data, 6 DEPs were chosen for validation by Western blotting. These DEPs included putative proteasome subunit alpha type-2, 3-ketoacyl-CoA thiolase 1, tropomyosin-2, hypothetical protein VM1G_00567, hypothetical protein VM1G_08430, and hypothetical protein VM1G_02622. As shown in Fig. 10, all six DEPs had higher expression levels in high pH (pH 6.0) samples as compared to the low pH (pH 3.4) samples, consistent with the proteomics results. These findings demonstrate the reliability and consistency of the proteomics data.

### Identified pathogenicity-related and virulence proteins regulated by pH and *VmPacC*

To identify pathogenicity-related proteins and virulence proteins that are regulated by pH and *VmPacC* in *V. mali*, the 119 proteins shared between the PacC versus WT groups and the pH 6.0 versus pH 3.4 groups were compared with the pathogen-host interaction database (PHI-base) v.4.13 (http://www.phi-base.org/) (Winnenburg et al. 2006) using the BLASTp algorithm. Interestingly, this analysis revealed that more than half (67/119) of the shared proteins had significant similarity (e-value, 10^–5^) with some of the genes listed in the PHI-base, suggesting that these unique proteins may be potential virulence factors and may play an important role in pathogenesis.

## Discussion

Proteomic analysis provides a large amount of information regarding the individual proteins that are involved in specific biological responses (de Oliveira and de Graaff 2011). In this study, TMT technology was used to analyze the proteomic changes in *V. mali* due to *VmPacC* deletion and under conditions of different ambient pH. The results indicated that numerous DEPs were involved in response to environmental pH. Among these DEPs, many proteins were regulated by *VmPacC* and were linked to diverse metabolic processes.

### DEPs involved in the *V. mali* pH response

The PacC signaling pathway is a specialized pH response pathway that has been extensively studied in multiple fungal species due to its crucial role in fungal pathogenesis. In this study, DEPs from apple twigs infected with the wild-type strain and the *VmPacC* deletion mutant strain highlighted the significance of the PacC signaling pathway in the interaction between *V. mali* and its host. However, as demonstrated in Fig. 4B, out of the 921 pH-responsive proteins, only 119 proteins were regulated by *VmPacC*. This led us to speculate that the pH regulation does not solely reply on the PacC signaling pathway, and suggests the existence of a pH-sensing mechanism other than the PacC pathway. Furthermore, the PacC signaling pathway works in collaboration with other signaling pathways to modulate cellular functions in response to changes in pH.

### DEPs associated with pathogenicity

Numerous studies have reported that the signal transcription factor PacC significantly influences pathogenicity by regulating the expression of various virulence factors of phytopathogenic fungi. For example, in *C. gloeosporioides*, pacC knockout mutants exhibited a decrease in pectate lyase secretion and significantly reduced virulence attributed to reduced *pelb* gene expression (Miyara et al. 2008). In *P. expansum*, PacC regulated the expression of the virulence factor glucose oxidase (GOX), calreticulin (CRT), and sulfate adenylyltransferase (SAT) (Chen et al. 2022). Here, we identified many DEPs regulated by *VmPacC*. We speculated that these proteins regulated by *VmPacC* were directly or indirectly involved in pathogenicity of this pathogen. For instance, among these DEPs, a subtilisin-like protease spm1 (KUI69588.1) was significantly up-regulated in the PacC-V group relative to the healthy control PacC-H. The ortholog of KUI69588.1 has been shown to be involved in the pathogenicity of a variety of fungal pathogens such as *M. oryzae* (Saitoh et al. 2009), *Cryphonectria parasitica* (Shi et al. 2014), *Alternaria alternata* (Fu et al. 2020), *Botrytis cinerea* (Liu et al. 2020), *Ustilaginoidea virens* (Chen et al. 2022). Another of the PacC-V DEPs, ribonuclease T2-like proteins (KUI72745.1), has an ortholog, FoRnt2, that was found to be required for the virulence of *F. oxysporum* and to enhance plant susceptibility to pathogens and promote infection in plants (Qian et al. 2022). In addition, we also identified several other proteins including pectate lyases, aspartate aminotransferase, hexokinase, that could be pathogenicity factors regulated by pH and *VmPacC* in *V. mali*. The homologs of these proteins have been reported to contribute to the pathogenesis for other pathogens (Ben-Daniel et al. 2012; Wang et al. 2016; Rui and Hahn 2007). In summary, our study has identified several potential pathogenic proteins, whose functions need to be determined in future functional experiments that lead to a better understanding of the pathogenic mechanism of *V. mali*.

### DEPs associated with carbohydrate and energy metabolism

Carbohydrate and energy metabolism, including TCA cycle, pentose phosphate pathway, glycolysis/gluconeogenesis, starch and sucrose metabolisms, is one of the most important regulators for a fungus to adapt to abiotic stress (Wang et al. 2013). Under adverse conditions, the TCA cycle becomes an important protective system, and the increase in the TCA cycle helpes the fungus cope with adverse conditions (Li et al. 2018). In this study, several DEPs, such as citrate synthase (KUI73686.1), pyruvate kinase (KUI70094.1), malate dehydrogenase (KUI64431.1), aconitate hydratase (KUI72241.1) and phosphoglucomutase (KUI66186.1), were significantly up-regulated at pH 6.0, which showed that the TCA cycle was activated and provided more energy for *V. mali* cells to deal with ambient high pH. The simultaneous up-regulation of proteins involved in glycolysis and the pentose phosphate pathway also reflect that the conversion of nutrient reserves to usable energy to drive the biosynthetic processes are needed for the fungus to cope with abiotic stress. A key protein enriched at pH 6.0 is the plasma membrane ATPase (KUI73682.1), which generates a proton gradient across the cell membrane to provide for the energy production required for the transportation of nutrients from the environment (Szabo and Bushnell 2001). In addition, previous studies have shown that glutathione metabolism also plays an important role in the fungal stress response. Our proteomic analysis revealed that two GSTs proteins (KUI71221.1 and KUI67220.1) involved in glutathione metabolism were up-regulated under high pH, suggesting that this pathway may be activated and plays an important role in the process of acidifying the environmental pH.

### DEPs associated with amino acid biosynthesis and metabolism

Amino acid biosynthesis and metabolism are very important for fungi during development and for the stress response. These processes involve major long-distance nitrogen transport carriers in fungal systems, important metabolites, molecular forms of nitrogen storage, and stress response signal-transduction molecules (Marty et al. 2019). The balance between protein synthesis and degradation plays a crucial role in regulating biological cell processes and their responses to developmental or environmental cues (Hasan et al. 2022). Our results revealed DEPs enriching arginine biosynthesis; lysine biosynthesis and degradation; as well as cysteine, methionine, alanine, aspartate, and glutamate metabolism. Aspartate aminotransferase (AST), in particular, plays a key role in the metabolic regulation of C- and N-metabolism in micro-organisms. The aspartate levels in the interior of micro-organisms are particularly important for its role as a starting point in the biosynthesis pathways for multiple amino acid. In turn, these amino acids are precursors of numerous metabolites playing distinct roles in the organisms’ growth, reproduction, development, or stress (Liu et al. 2017). We found that two AST (KUI73864.1 and KUI68644.1) that are involved in amino acid biosynthesis and metabolism were significantly up-regulated at pH 6.0, when compared for with pH 3.4. These two DEPs may play important roles in the stress induced by environment pH changes.

### DEPs associated with ribosome

Ribosomal proteins are involved in the synthesis of new ribosomes with ribosomal RNA, but they also function in vitro to regulate gene transcription and cell proliferation, differentiation, and apoptosis. We found that most of the ribosome-related DEPs were up-regulated at pH 6.0, indicating that the biogenesis of ribosomes in *V. mali* is activated. Thus, the enhanced ribosomal function during regualtion to high pH environments of *V. mali* is likely helpful for increasing the expression of pathogenicity-related genes.

### PacC-mediated pH regulation signaling pathway in *V. mali*

**Fig. 11:**
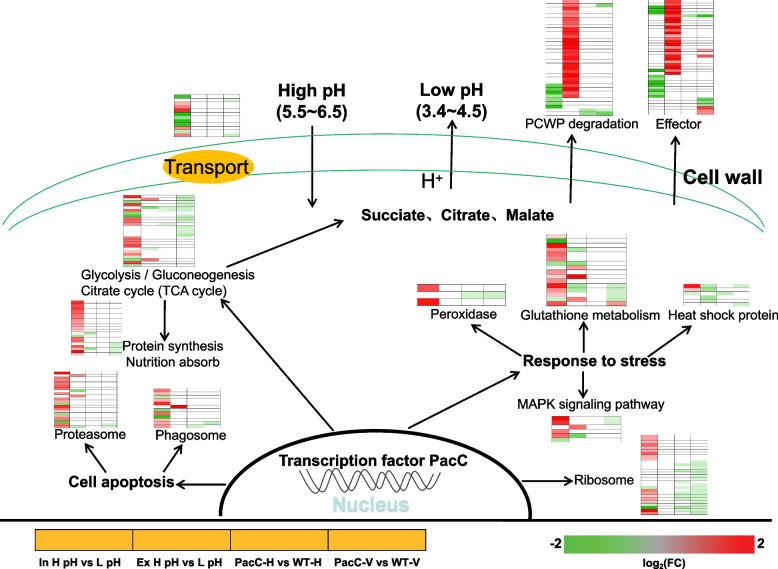
Model of the hypothetical *V. mali* response to ambient pH. H, healthy tissue; V diseased tissue; ‘In’, intracellular; ‘Ex’; extracellular; H-pH, pH 6.0; L-pH, pH 3.4

Based on the above results, we proposed a PacC-mediated pH regulation signaling network in *V. mali* (Fig. 11). The optimum pH of *V. mali* is about 3.5 (Wu et al. 2018). When the external pH is neutral or alkaline, the pathogen senses the low concentration of hydrogen ions and produces acidic substances through a series of complicated cellular biological processes to maintain the intracellular pH homeostasis and/or change the ambient pH. The biological signal produced by the difference in the concentrations of extracellular and intracellular hydrogen ions activates the pH-responsive transcription factor PacC. Thus, PacC participates in the process of pathogen regualtion to ambient pH. Both hydroxyl ion and PacC activate and regulate the MAPK signal pathway, then send signals to induce glutathione, heat shock protein, and peroxidase production to help avoid adverse environmental conditions.

When ambient pH is not suitable for the growth of *V. mali*, differences in the ion concentration and PacC together regulate the ribosome pathway, which can induce the expression of many ribosomal genes, which then help the pathogen to produce more proteins to cope with the adverse environment. Notably, the glycolysis/gluconeogenesis pathway and TCA cycle are the main pathways that provide energy for the organisms in case of excessive energy consumption under the adverse external environmental conditions. Moreover, genes involved in the above two pathways of *V. mali* are in an active state, thereby assuring increased production of energy and organic acids, such as citric acid, malic acid, and succinic acid, ultimately changing the extracellular pH to a favorable one.

## Conclusion

We investigated *VmPacC-*mediated pH regulation in *V. mali* by comparing the DEPs of *ΔVmPacC/WT* and HpH/LpH and found altered accumulation of numerous proteins. A GO and KEGG pathway analysis indicated that *VmPacC* participated in the pathogen response to ambient pH via regulating the energy metabolism pathway. We propose a pH regulation model that accounts for the mechanism of *V. mali* regulation to ambient pH, which we hope will guide future research to identify potential molecular targets for new pesticides and to accelerate the prevention and control of this important disease.

## Materials and methods

### Fungal strains and culture conditions

The wild-type *V. mali* strain 03-8 and *VmPacC* deletion mutant *ΔVmPacC* were obtained from the Laboratory of Integrated Management of Plant Diseases at the College of Plant Protection, Northwest A&F University, Shaanxi, Yangling, China. The strains were recovered from 30% glycerol solution at -80℃, and then grown on potato dextrose agar (PDA, 20% potato extract, 2% dextrose, 1.5% agar) (Wu et al. 2018).

### Sample preparation

To generate samples for the proteomics analysis of fungus during infection, wild-type strain 03-8 and *VmPacC* deletion mutant were cultured on PDA medium for 2 d. Then 5-mm agar plugs were taken from the edge of a colony and were used to inoculate the scald wounds made on 1-year old twigs of *Malus domestica borkh*. ‘Fuji’ at 25℃ for 3 d (Zang et al. 2007). Then 0.2 g samples of healthy and diseased twig tissues were collected and frozen in liquid nitrogen for storage at -80℃ until further use.

To perform the proteomics analysis of *V. mali* under different pH conditions, samples of the wild-type *V. mali* strain 03-8 were grown for 48 h on PDA medium on the surface of cellophane membranes inoculated with 5-mm mycelial plugs, and then transferred to Yeast Extract Peptone Dextrose Medium (YEPD, 0.3% yeast extract, 1% peptone, 2% glucose), buffered at pH 6.0 or pH 3.4 by using 0.2 M Na_2_HPO_4_ and 0.1 M citric acid to maintain pH, and continued to grow for 1 d. Mycelia were collected using two layers of filtration fabric and washed three times with distilled water. The mycelium and filtrates were then quickly frozen with liquid nitrogen and stored at -80℃ until further use. For each condition, three independent biological replicates were prepared and assayed.

### Protein extraction

The samples collected from the healthy or diseased tissues were ground into cell powder by using liquid nitrogen and then transferred into 5-mL centrifuge tubes and mixed with four-fold volumes of lysis buffer (8 M urea, 1% Triton-100, 10 mM dithiothreitol, and 1% protease inhibitor cocktail). The mixture was sonicated three times on ice using a high intensity ultrasonic processor (Scientz, Ningbo, China). The remaining debris was removed by centrifugation at 12,000 × g for 10 min at 4℃. Finally, the protein was precipitated with 20% TCA at -20 ℃ for 2 h. After centrifugation at 12,000 × g for 10 min at 4℃, the supernatant was discarded. The remaining precipitate was washed with cold acetone for three times. The protein was re-dissolved in 8 M urea and the amount of protein was quantified by using the BCA Protein Assay Kit (TIANGEN, Beijing, China) (Catalog number: PA115-01) according to the manufacturer’s instructions.

To harvest secreted proteins of *V. mali* under different pH conditions, the filtrates were centrifuged at 8000 × g for 15 min at 4℃. Then, the supernatant was transferred to a new ultrafiltration centrifugal tube (Merck Millipore; Amicon Ultra-50, Ultracel-3 k, Germany) and was centrifuged at 5000 × g for 2 h at 4 ℃. Next, an equal volume of Tris-saturated phenol (pH 8.0) was added to the concentrates, and the mixture was centrifuged at 5500 × g for 10 min at 4℃. The secreted proteins were precipitated with five volumes of 0.1 M ammonium acetate-saturated methanol incubated at 4℃ overnight. The supernatant was discarded after centrifugation at 12,000 × g for 10 min at 4℃. The remaining precipitate was washed with cold methanol and acetone for three times. The protein was re-dissolved in 8 M urea and quantified by using the BCA Protein Assay Kit. The experimental procedure for the extraction of mycelium proteins was the same as the extraction from the healthy or diseased tissues. Three independent biological replicates were prepared and assayed for each sample.

### Protein digestion and TMT labeling

Small samples (100 μg) of crude proteins prepared from each tissue sample were condensed with 5 mM dithiothreitol (DTT) for 1 h at 65 ℃, and then alkylated with 11 mM iodoacetamide (IA) for 30 min at room temperature in darkness. The protein sample was then diluted by adding 100 mM triethylammonium bicarbonate (TEAB) to urea concentration less than 2 M. Finally, freshly prepared trypsin (Trypsin Gold, Mass Spectrometry Grade, Promega) was added at a 1:50 w/w trypsin/protein ratio for the overnight protein digestion at 37 ℃.

The tryptic peptides resulting from trypsin digestion were vacuum concentrated and re-suspended in 0.5 M TEAB and processed for labeling using the TMT Mass Tagging Kits and Reagents according to the manufacturer’s protocol (Thermo Fisher Scientific, USA). Briefly, one unit of TMT reagent (Catalog number: 90060) was thawed and reconstituted in anhydrous acetonitrile. The peptide mixtures were then incubated for 2 h at room temperature, pooled, desalted using C18 spin tips (Thermo Fisher Scientific), speed-vacuum dried, and stored at -80 ℃ prior to LC-MS/MS analysis.

### Nano LC-MS/MS analysis

The protein samples were identified by using shotgun analysis (Shanghai Applied Protein Technology Co. Ltd., Shanghai, China). Labeled peptides from eight protein libraries were mixed and dissolved in buffer A (0.1% formic acid), and then were loaded on a reverse-phase trap column (Acclaim PepMap100, 100 μm × 2 cm, nanoViper C18; Thermo Fisher Scientific) connected to a C18-reverse-phase analytical column (Easy Column, 10 cm length, 75 μm inner diameter, 3 μm resin; Thermo Fisher Scientific). The Buffer B gradient (in a solvent containing 0.1% formic acid in 98% acetonitrile) was gradually increased from 3 to 23% over 26 min, then increased from 23 to 35% over 15 min, then climbed from 35 to 80% in 3 min, and finally maintained at 80% for 3 min at a constant flow rate of 300 nL/min controlled by IntelliFlow technology (Thermo Fisher Scientific).

The separated peptides were ionized using a nano-spray ionization source (electrospray voltage, 2.0 kV), followed by tandem mass spectrometry (MS/MS) in Q Exactive™ Plus (Thermo Fisher Scientific) coupled online to UPLC. Using full scan range (350–1800 m/z) in MS, peptides were picked out for MS/MS with NCE setting at 30, and fragments were detected in the Orbitrap at a resolution of 17,500. A data-dependent procedure alternated between one MS scan and 20 MS/MS scans with 15 s dynamic exclusion. Fixed first mass was set to 100 m/z. Automatic gain control (AGC) was set to 5 × 10^4^.

### Protein identification and quantification

The raw data were processed using MaxQuant (v.1.5.2.8), and database searches were carried out using the *V. mali* protein database (NCBI). Trypsin/P was specified as cleavage enzyme, with a threshold of two missed cleavages. The precursor mass tolerance was set at 20 ppm, with a fragment ion mass tolerance of 0.02 Da. Carbamidomethyl (C) oxidation was specified as static modification, and methionine (M) oxidation was specified as dynamic modification. A decoy database search strategy was used to determine the false discovery rate (FDR) for peptide and protein identification. Peptide identifications were accepted with FDR < 0.01, while protein identifications contained at least one identified peptide. Relative fold changes (FC) is achieved by comparing the MS/MS intensity of the specific peptide of the protein. The mass spectrum intensity of specific peptide was the MS/MS intensity of the corresponding TMT label group. Relative FC calculated for the comparison of two groups, a significantly up-regulated protein (with a FC > 1.3, *p* < 0.05) or down-regulated protein (FC < 0.77, *p* < 0.05) was considered as a DEP.

### Bioinformatics and statistical analyses

To investigate the biological functions of the identified DEPs, we annotated the functions of these proteins by using gene ontology (GO) analysis. The GO annotation proteome was derived from the UniProt-GOA database (https://www.ebi.ac.uk/GOA/). First, the ID of the identified protein was converted to a UniProt ID, which was then mapped to GO IDs by the ‘protein ID’ function. For identified proteins that were not annotated in the UniProt-GOA database, the InterProScan software (http://www.ebi.ac.uk/interpro/) was used to annotate each protein’s GO function. The GO terms were considered significantly different for *p* < 0.05. Finally, based on the GO annotation, the proteins were classified according to three categories: biological processes (BP), cellular components (CC), and molecular functions (MF).

For pathway annotation, we used the KEGG online service tool KAAS (http://www.genome.jp/kaas-bin/kaas_main) to annotate each protein in KEGG database. Next, to map the KASS annotation to the KEGG pathway database, we used the online service tool ‘KEGG mapper’ (http://www.kegg.jp/kegg/mapper.html). We considered a KEGG pathway significantly enriched for *p* < 0.05. Subcellular localization of the DEPs was predicted using Wolfpsort (V.0.2) (http://www.genscript.com/psort/wolf_psort.html).

Lastly, the protein-protein interaction (PPI) networks were investigated using the STRING database (https://cn.string-db.org/) to visualize the distribution characteristics of partial DEPs, which included direct and indirect associations of proteins. STRING defines for each interaction a measure of confidence, called the confidence score; we included all interactions with a medium or higher confidence (low confidence - 0.15, medium confidence - 0.4, high confidence - 0.7, highest confidence - 0.9). The resulting PPI network graph was generated for the selected DEPs using Cytoscape software (http://www.cytoscape.org/, v.3.1).

### Transcriptional expression pattern of some DEPs in response to different pH conditions

The wild-type *V. mali* strain 03-8 was inoculated onto PDA medium covered with a layer of cellophane membrane at 25 ℃ for 2 days. The membranes were transferred onto PDA plates buffered at pH 6.0 or pH 3.4. Mycelia were collected at 0, 2, 4, 6, 8 post-inoculation hours (hpi) and frozen in liquid nitrogen and stored at -80℃ for RNA extraction at a later time.

Total RNA was extracted from each collected sample using the Quick RNA isolation Kit (Huayueyang, Beijing, China) according to the manufacturer’s protocol. First strand cDNA was synthesized using the RevertAid First Strand cDNA Synthesis Kit (Thermo Fisher Scientific). The qRT-PCR was carried out using 2 × RealStar Power SYBR Mixture (GenStar, Beijing, China). Glucose 6 Phosphate Dehydrogenase (*G6PDH*) of *V. mali* was chosen as the endogenous reference gene (Table 1) (Yin et al. 2013). Finally, the relative expression levels were calculated according to the 2^−*ΔΔCT*^ method (Livak and Schmittgen 2001) as the results of detection. The entire analysis was performed three times (on three independent biological replicates).

**Table 1:** Primers used for the qRT-PCR amplification of target genes

Protein accession	Protein name	Gene locus	Primer sequence (5 to 3_x005f)	Target Position	Size (bp)
KUI74097.1	Versicolorin reductase	VM1G_09945	F:GGCTCATCCTCACCTCGTCC	470–489	198
			R:CGTAATGCCAGGCGTTCTCA	667–648	
KUI73952.1	Acetyl-CoA hydrolase	VM1G_09468	F:TGCCAAGGGTATCAACGA	422–439	276
			R:GGAAGCACCAGGGACAAT	697–678	
KUI73686.1	Citrate synthase	VM1G_09382	F:CGATTGTCCCGAGTTTGATT	160–179	294
			R:CCCAGACGAGGCACTTGATA	453–434	
KUI65737.1	Arginase	VM1G_02398	F:CCCGATTCATCTGTCGTTTG	1002–1021	213
			R:CTGACCGTCTCGTTAGCACC	1214–1195	
KUI63718.1	Phospholipase D2	VM1G_10533	F:GGCGGCAATGCGGTCTAAAT	987–1006	230
			R:GCCTCAGAAAGAGCCCAGAAGTAA	1216–1193	
KUI73579.1	Heat shock protein 60	VM1G_09272	F:CGATTTAGACCCTTCTTTGC	123–142	183
			R:GACTCAATCAGGACGTTTCG	305–286	
KUI67500.1	Beta-cyclopiazonate dehydrogenase	VM1G_03091	F:GTTACGGTGGCACAGGACTATTT	4019–4041	259
			R:GGGATTGGAGGTTGTATTTGGT	4277–4256	
KUI65912.1	Glucose-6-phosphate 1-dehydrogenase	VM1G_01712	F:GCGGGTGTCTTGTGGACTAAC	194–214	118
			R:TCGCTCACGGTCTTCTTCTT	311–292	
KUI67090.1	Putative NADPH-dependent methylglyoxal reductase GRP2	VM1G_03315	F:GGCGAATGACACCATCCCTG	750–769	125
			R:CTGCCGTCGTGAACAACCTG	874–855	
KUI64990.1	Aldehyde dehydrogenase	VM1G_00680	F:ACATCCAGTCGGCGAAAGAA	2472–2491	280
			R:TCGATCTCAGCCGCCACTCT	2751–2732	
AGG40756.1	6-phosphogluconate dehydrogenase	G6PDH	F:ACTCCAACCGCAGGACCCAATA	34–55	120
			R:TCTCGTCACCACCAGGCATCAG	153–132	

### Gene overexpression

Initially, the full-length sequences of the 6 DEPs with their native promoters were amplified using standard PCR conditions. The resulting product was then ligated to pDL2-HA vector that had been digested with *XhoI*. The constructs were subsequently introduced into chemically competent *Escherichia coli* cells. After selection with selective antibiotics, individual colonies that had been verified by PCR were cultured in lysogeny broth medium at 37℃ in a shaking incubator at 220 rpm for 16 h. Finally, these constructs were transformed into protoplasts of the wild-type *V. mali*  strain 03-8.

### Western blot analysis

To validate the proteomics results, a Western blot was conducted. Firstly, the proteins from each group were extracted and separated using 12% SDS-PAGE and transferred to a polyvinylidene fluoride (PVDF) membrane using a semi-dry transfer cell. The membrane was rinsed consecutively in Tris buffered saline (TBS) and 5% skimmed milk in TBS containing 0.5% Tween 20 (TBST) for 2 h at room temperature with 50 rpm shaking, followed by incubated with 1:2500 diluted mouse anti-HA (Catalog number:26D11, Abmart, Shanghai, China) as the primary antibody at room temperature for 3 h. After being washed three times with TBST, the membranes were incubated with goat-anti mouse IgG (1:2500) as the secondary antibody at room temperature for 1 h. After washing three times with TBST, the color development was performed using enhanced chemiluminescence (ECL) substrate (Solarbio, Beijing, China) in accordance with the manufacturer’s instructions. Finally, the PVDF membranes were stained with Commassie blue (CBB) to verify that the loading amounts were equal.

## Supplementary Information

**Additional file 1: Figure S1.** The diagram of sample collection.

**Additional file 2: Figure S2.** Functional annotation of the 119 shared proteins in the PacC versus WT and the pH 6.0 versus pH 3.4 comparison groups.

**Additional file 3: Table S1.** The proteins shared between the PacC-H and WT-H groups, and between the PacC-V and WT-V groups. Green represent shared proteins.

**Additional file 4: Table S2.** The gene ontology (GO) annotation analysis of differentially expressed proteins (DEPs) between the PacC and WT groups.

**Additional file 5: Table S3.** The KEGG enrichment analysis of differentially expressed proteins (DEPs) between the PacC and WT groups.

**Additional file 6: Table S4.** The list of 921 pH-responsive proteins.

**Additional file 7: Table S5.** The 119 proteins shared between the PacC and WT groups and between the pH 6. 0 and pH 3.4 groups.

**Additional file 8: Table S6.** The 27 DEPs overlapped between the identified DEP lists with the co-expression network from the transcriptome profiling data (Ke et al. 2014).

## Data Availability

All data generated or analyzed during this study are included in this published article.

## Abbreviations

TMT: Tandem mass tag
LC-MS/MS: Liquid chromatograph-mass spectrometer
DEP: Differentially expressed protein
GO: Gene Ontology
KEGG: Kyoto Encyclopedia of Genes and Genomes
TCA cycle: Tricarboxylic acid cycle
PPI: Protein-protein interaction
PDA: Potato dextrose agar
YEPD: Yeast Extract Peptone Dextrose
TEAB: Triethylammonium bicarbonate
AGC: Automatic gain control
FDR: False discovery rate
FC: Fold changes
qRT-PCR: Quantitative reverse transcription polymerase chain reaction
NCBI: National Center of Biotechnology Information
G6PDH: Glucose 6 Phosphate Dehydrogenase
PHI-base: Pathogen-host interaction database
