# Supplementary figures and images for: VmPacC-mediated pH regulation of Valsa mali confers to host acidification identified by comparative proteomics analysis
Source: Stress Biol. 2023 Jun 21;3(1):18. doi: 10.1007/s44154-023-00097-y (PMC10441875; doi:10.1007/s44154-023-00097-y)

Figure S1

WT                       $\Delta VmPacC$

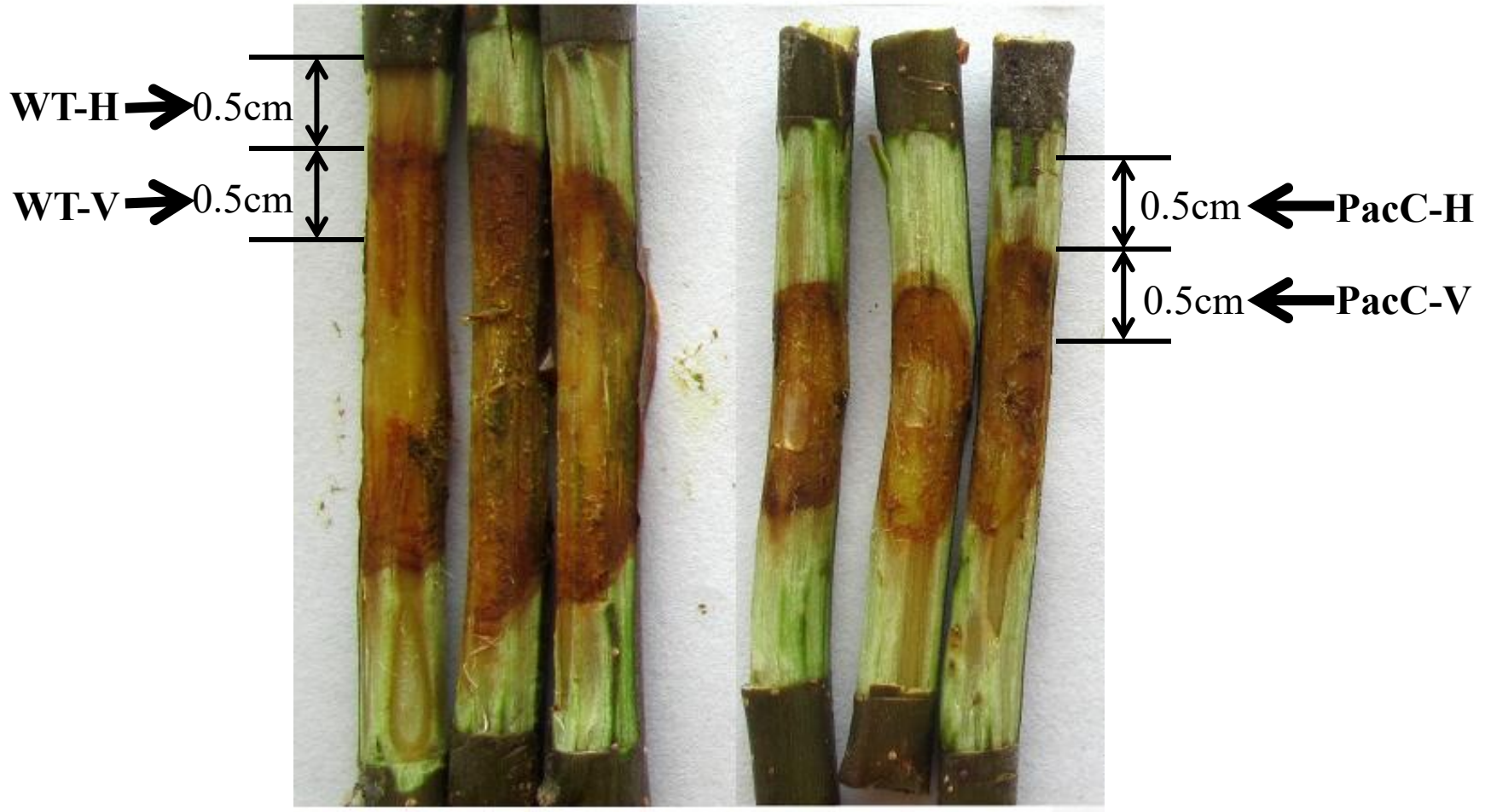

Supplement: Supplementary file 1 — Additional file 1: Figure S1. The diagram of sample collection. [file 44154_2023_97_MOESM1_ESM.pdf]

**Figure S2**

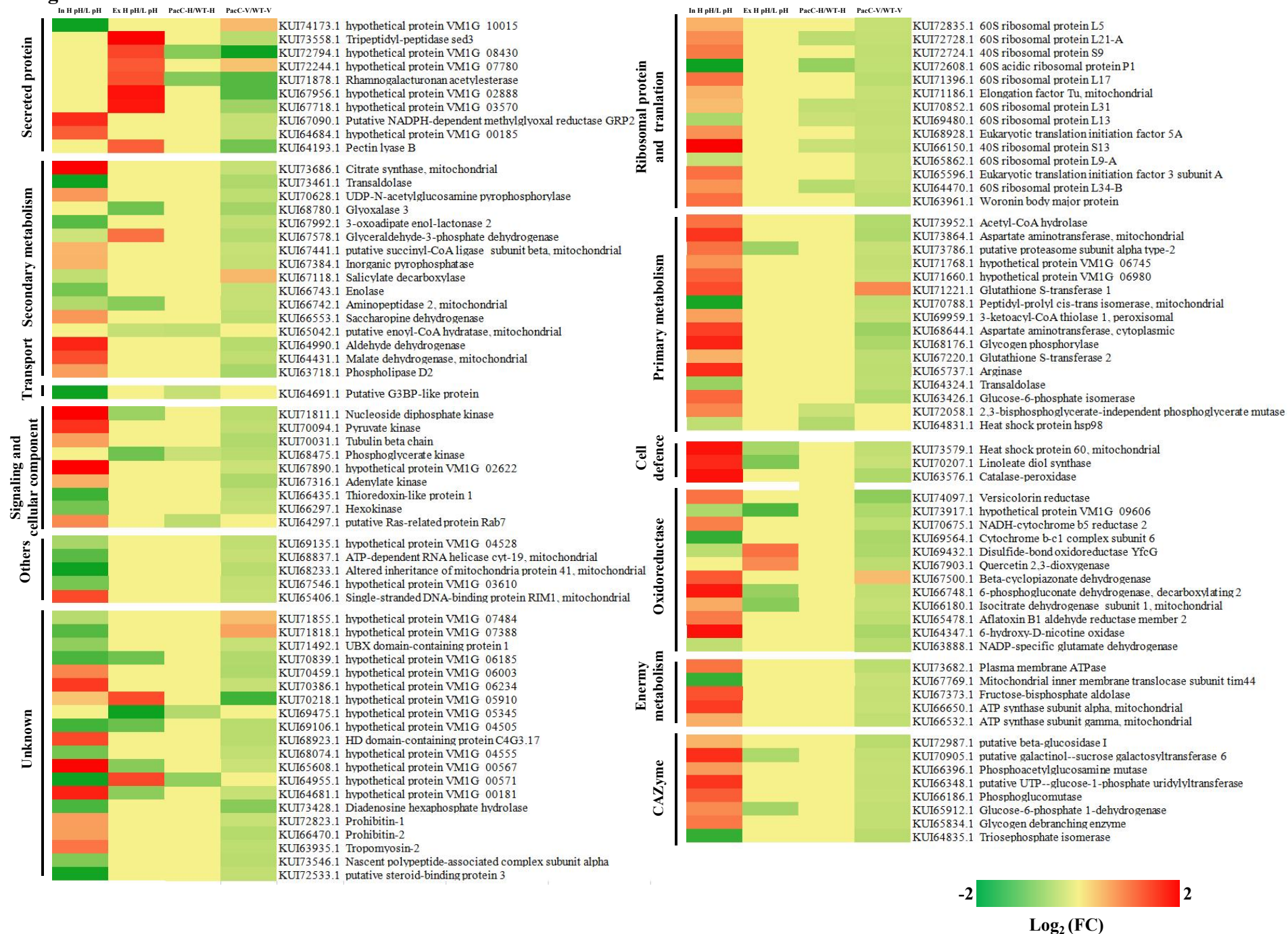

Supplement: Supplementary file 2 — Additional file 2: Figure S2. Functional annotation of the 119 shared proteins in the PacC versus WT and the pH 6.0 versus pH 3.4 comparison groups. [file 44154_2023_97_MOESM2_ESM.pdf]
